# Our Bureau of Information

**Published:** 1911-12-30

**Authors:** 


### OUR BUREAU OF INFORMATION.

### Brotherhood Work.

### Hospital Entertainments.

At Christmas time most of our hospital wards are gay, there is singing of carols, sometimes a Christmas tree—at least for the children—and often gifts for the older patients. Most of the festivities are got up by the nurses and the resident staff, and as these have already enough to do, it may be forgiven them if they sometimes say "Thank goodness!" when the festivities are over. But "Christmas comes but once a year," and there are three hundred and sixty-four days beside. Some of these days are long and dreary, and might be made brighter both for patients and nurses if outsiders would bring to them some of the entertainments which they cannot go out to seek. To a nurse, a visit to theatre or concert must needs be a very rare treat. Patients, accustomed before their illness to pay an occasional visit to theatre, music-hall, or cinematograph, may find, when the first acute stage of sickness is past, that the days are dull and long. No good comes of dulness, and though there are certainly times in the course of illness when excitement is bad, there are also times when a little change is a healthful stimulus.

In general hospitals, where the average stay is twenty-one days, there may be no pressing need for entertainment—though it is well to remember that even there the average is often exceeded—but in hospitals for incurables admission is always a "life sentence," and the life is often prolonged, while in others, such as hospitals for consumption and cancer, the patient's stay is almost always a long one. In all such institutions entertainments provided by outside friends are welcomed by the authorities, and in these days when every suburb and almost every village has its dramatic club and its choral society, there is no reason why these societies should not give a performance to the patients of the nearest hospital. In some hospitals such performances are a regular feature of the winter months, and when they are offered there is very seldom any difficulty made about fitting up an out-patient department or an—alas!—empty ward to serve the temporary needs of the performance. Some institutions, indeed, have apartments with a regular stage, but even if this be lacking a very good entertainment can be given if care is taken to choose only plays that do not need elaborate staging. Concerts, of course, are an even simpler matter, and are always appreciated, while, if there is anything of the nature of a stage or platform, dancing—morris, national, or classical—is sure of a rapturous welcome.

It is well to accentuate the fact that entertainments in any institution which shelters the sick, the poor, or the distressed, should be of a cheerful nature. There is an idea among many people that entertainments for the poor and the suffering must always be of a serious and even of a solemn kind. But as on the earth we have day and night, sunshine and shadow, so in life we need laughter as well as tears, bright thoughts as well as serious ones. And when circumstances force one to look on the solemn side of things it is good to be reminded of their more joyous aspect. The religious welfare of the inmates of all our institutions is looked after, whatever their creed, by competent and earnest men of their own denomination, and though the singing of some of those hymns which Christians of all sects unite in loving may be done in the wards with acceptance, it should never be forgotten that any discursions into theology are apt to be unwise. The spirit of true religion is in all work for the sick and the poor, and will have its leavening influence without more insistence on creed than is supplied by the usual religious services.

But our patients have their human sympathies, their natural liking for human mirth and beauty, which those outside the institution may well cater for. Even where no more formal entertainment can be arranged for, any woman with a sweet voice may do a little deed of kindness by singing in the wards. Old songs and new are heard with pleasure, though, perhaps, the best loved are those old-fashioned ones that everyone knows almost without remembering having learnt them—such songs as "Home, Sweet Home," "The Last Rose of Summer," and "Annie Laurie."

We have spoken of entertainments in hospitals, but all that we have said applies equally to workhouses and asylums. Life is a very hopeless thing in the workhouse, and any break in its monotony is welcomed, and in them there is always the big dining-hall which can be utilised as concert-room. We have heard a very good—and much appreciated—rendering of Gilbert and Sullivan's "Trial by Jury" in a workhouse. In asylums there are usually proper recreation-rooms with stage and all the necessaries for dramatic performances at command, for those in authority know that depression is a serious factor in mental disease. But depression is a factor in all sickness and in all lives that have lost the stimulus of hope, and outsiders who know no other way of giving help may often do good service by brightening for an hour or two the lives of those who either temporarily or permanently have fallen by the way. The apostle's words hold both instruction and encouragement: "Silver and gold have I none, but such as I have give I thee."

### How to Make the Bureau a Success.

Everyone who uses or wishes to help our Bureau of Information must be kind enough to observe the following rules:

(1) Postal replies cannot be given. Exception will only be made in very special cases at the Editor's discretion.

(2) Queries or answers relating to different cases must be written, or preferably typed, on separate sheets of paper, and the writing must never be crossed.

(3) Questions and replies received not later than Wednesday will, as far as possible, be dealt with in our issue of the following Saturday week.

(4) Letters must have attached the name and address of the sender, with a pseudonym for publication if preferred.

(5) All applicants for insertion in our list of Approved Homes must send a registration fee of five shillings. The omission of this leads to delay in the insertion of names.

(6) *All communications* for this department *must be accompanied by the coupon* to be found at the bottom of the third page of the cover on the inside, and should be addressed to the Editor of the Bureau of Information, c/o The Hospital, 29 Southampton Street, Strand, London, W.C.

Notice.—We invite workers of special experience and knowledge to offer help in replying to questions relating to their special department.

### Needs and Helpers.

"R. Rochdale" writes: I would like to hear of a Private Asylum or Home for a young lady of twenty-four somewhere in Lancashire—neighbourhood of Southport preferred. She is strong physically, and would be the better of some duties to occupy her, but would need control, as she is quite wrong mentally.

"Hope" writes: I should like to take entire and permanent care of a child a year old or more. I am the widow of an officer in the Royal Navy, and have a pleasant country home. Terms, £1 per week, to include everything—board, lodging, clothing, and education when required. I should like to have the child entirely given over to me, as I should not like to part with the child when I have grown fond of it.

